# The use of standardized Brazilian green propolis extract (EPP-AF) as an adjunct treatment for hospitalized COVID-19 patients (BeeCovid2): a structured summary of a study protocol for a randomized controlled trial

**DOI:** 10.1186/s13063-022-06176-1

**Published:** 2022-04-04

**Authors:** Marcelo Augusto Duarte Silveira, Sergio Pinto de Souza, Erica Batista dos Santos Galvão, Maurício Brito Teixeira, Marcel Miranda Dantas Gomes, Lucas Petri Damiani, Bruno Andrade Bahiense, Julia Barros Cabral, Cicero Wandson Luiz Macedo De Oliveira, Talita Rocha Mascarenhas, Priscila Carvalho Guedes Pinheiro, Milena Souza Alves, Rodrigo Morel Vieira de Melo, Andresa Aparecida Berretta, Flávia Mendes Leite, Carolina Kymie Vasques Nonaka, Bruno Solano de Freitas Souza, Ana Verena Almeida Mendes, Suzete Farias da Guarda, Rogério da Hora Passos

**Affiliations:** 1 D’Or Institute for Research and Education (IDOR), Hospital São Rafael, Avenida São Rafael 2152, São Marcos, Salvador, BA 41253-190 Brazil; 2 HCor Research Institute, São Paulo, Brazil; 3 School of Medicine, Federal University of Bahia, Salvador, Bahia Brazil; 4 Research, Development and Innovation Department, Apis Flora Indl. Coml. Ltda, Rua Triunfo 945, Subsetor Sul 3, Ribeirão Preto, SP 14020-670 Brazil; 5 Gonçalo Moniz Institute, Oswaldo Cruz Foundation (FIOCRUZ), Salvador, BA 40296-710 Brazil

**Keywords:** COVID-19, Randomized controlled trial, Protocol, Propolis, Anti-inflammatory agents, Immunoregulation, ACE2, PAK1 blocker, TMPRSS2

## Abstract

**Background:**

The 2019 coronavirus disease (COVID-19) pandemic continues to spread and affects large numbers of people with unprecedented impacts. Experimental evidence has already been obtained for use of the standardized extract of Brazilian green propolis (EPP-AF) against viral targets, and clinical rationality has been demonstrated for testing this extract as an adjunct to treatment in patients affected by COVID-19. The BeeCovid2 study aims to assess whether EPP-AF has an impact on the improvement of patients hospitalized with COVID-19 by reducing the length of hospital stay.

**Methods:**

BeeCovid2 is a randomized, double-blinded, placebo-controlled clinical study being conducted in Brazil to provide further evidence on the effectiveness of standardized green propolis extract as an adjunctive treatment for adults hospitalized with COVID-19. Hospitalized patients over 18 years of age with a confirmed diagnosis of COVID-19 and up to 14 days of symptoms were included. Patients under mechanical ventilation at randomization, pregnant women, cancer patients, transplanted or using immunosuppression, HIV patients, patients who used propolis in the last 30 days, bacterial or fungal infection at randomization, impossibility of using medication orally or enterally, and advanced chronic diseases (e.g., advanced heart failure, severe liver disease, and end-stage chronic kidney disease). Enrolled patients are randomized at a 1:1 ratio to receive placebo or standardized propolis extract (900 mg/day) for 10 days. The study treatments are administered in a double-blinded manner, and patients are followed for 28 days. The primary outcome is the difference in length of hospital stay in days between groups. Secondary outcomes include the need for mechanical ventilation, the rate of secondary infection, rate of acute kidney injury, the need for renal replacement therapy, the requirement for vasoactive drugs, the use of an intra-aortic balloon pump (IABP), and the use of extracorporeal membrane oxygenation (ECMO).

**Discussion:**

This trial is very useful and will provide more data on the effectiveness of using the standardized Brazilian green propolis extract as an adjunctive treatment in association with standard care in adults hospitalized with moderate to severe acute COVID-19.

**Trial registration:**

ClinicalTrials.gov NCT04800224. Registered on March 16, 2021.

## Background

The COVID-19 pandemic is of great concern because of its unprecedented impact on human health [1]. The disease, after the replication phase, promotes inflammatory and immunological events, and even with advances in knowledge of its pathophysiology, we still do not have a specific treatment [1].

Coronavirus infection therefore presents immunological and inflammatory challenges for clinical practice. Experimental and clinical evidence points to greater infectivity and enhanced promotion of immune-inflammatory processes by coronavirus [1–3]. Further understanding and the search for strategies that concurrently impact both of these mechanisms can bring about a reasonable solution for patients affected by the disease, mainly because the response to these events is heterogeneous, and modulation of this response may be the best strategy [1].

Propolis is a natural resin with considerable evidence of antioxidant, antiviral, immunoregulatory and anti-inflammatory activities, and experimental data point to its potential actions against viral targets [4].

### Propolis: experimental evidence against viral targets

The coronavirus uses several mechanisms in the processes of activation, cell invasion, replication, and the triggering of inflammatory mechanisms [1–3].

The entry of SARS-CoV-2 into target cells requires binding of the spike protein to angiotensin-converting enzyme 2 (ACE2) [1–3, 5, 6]. The spike protein is first activated by the membrane protease human transmembrane protease 2 (TMPRSS2) [5]. After switching on, several signals are triggered, allowing for viral endocytosis and activation of the PAK1 inflammatory pathway, which reduces the adaptive immune response and the production of antibodies against the virus [4, 7]. The PAK1 pathway also promotes the activity of C-C pathway motif chemokine ligand 2 (CCL2), which is associated with stimulating pulmonary fibrosis [4, 7]. Further, macrophage and monocyte activation occurs, as well as production of additional cytokines, including TNF and IL-6 [1]. Compounds derived from green propolis negatively regulate the expression of TMPRSS2 and the anchoring of ACE2, which limits entry of the virus [4–6, 8]. Experimental evidence also points to propolis substances capable of reducing activation of the PAK1 pathway, an important target used by the virus to shield itself from adaptive immunity [7].

### Clinical evidence from the use of standardized green propolis extract for COVID-19

Clinical evidence has begun to emerge that has increased understanding of the therapeutic possibilities of using propolis to combat the coronavirus.

The first clinical study using propolis in patients with COVID-19 worldwide was recently published in the scientific literature [9]. It was randomized, controlled, and open and was conducted at Hospital São Rafael in Salvador, Bahia, Brazil. After approval by the National Research Ethics Committee in Brazil, it was registered on the clinicaltrials.gov platform (NCT04480593). Standardized Brazilian green propolis extract (EPP-AF) was used at two different doses (low and high doses). This study included 124 patients who were divided into three groups: the control group (*n* = 42), the EPP-AF group receiving 400 mg/day (*n* = 40), and the EPP-AF group receiving 800 mg/day (*n* = 42).

All patients received standard treatment that included the use of corticosteroids, antibiotics, antivirals, oxygen therapy, or any necessary support (e.g., extracorporeal membrane oxygenation [ECMO], intra-aortic balloon pump [IABP], dialysis). The study protocol did not interfere with decisions about supportive treatment. The primary outcome assessed was the time to disease recovery, inferred by length of hospital stay after randomization. The length of hospital stay postintervention was shorter in both propolis groups than in the control group: in the low dose group, the median stay was 7 days versus 12 days (95% confidence interval [CI] − 6.23 to − 0.07; *p* = 0.049); and in the high dose group, the median stay was 6 days versus 12 days (95% CI − 7.00 to − 1.09; *p* = 0.009) [two]. The patients who received EPP-AF at the highest dose had a significantly lower rate of acute kidney injury than those in the control group (23.8% vs. 4.8%, *p* = 0.048) [9]. The study had limitations: it was an open, single-center study and did not include a placebo group. The use of propolis at the studied doses was safe and beneficial, and the baseline characteristics of the participants were similar with regard to comorbidities, duration of illness, degree of lung involvement (as assessed by tomography), and oxygen therapy.

## Methods

### Design and oversight

BeeCovid2 is a randomized, double-blinded, placebo-controlled clinical trial being conducted at Hospital São Rafael, a tertiary hospital in Salvador, Bahia, northeastern Brazil. This study began on April 14, 2021, with the aim of including other centers.

The protocol was approved by the Local Ethics Committee (registration number 43265321.9.0000.0048) on February 25, 2021, and the trial was registered on March 16, 2021, on ClinicalTrials.gov (NCT04800224). The study is being conducted in accordance with the principles of the Declaration of Helsinki and the guidelines of Good Clinical Practice of the International Harmonization Conference. All participating patients and/or legal representatives are informed about the objectives and risks of participation before signing the informed consent form. The informed consent is electronically signed, stored on the Research Electronic Data Capture (RedCap) platform and automatically made available to participants via download. Electronic informed consent was appreciated and approved by the local ethics committee, and it offers more security to the research team (Table 1).

**Table 1:** Participant timeline

	Study period									
	Enrolment/allocation				Post-allocation					Close-out
Timepoint	April 2021	May 2021	June 2021	July 2021	April 2021	May 2021	June 2021	July 2021		August 2021
**Enrolment:**										
Eligibility screen	x	x	x	x						
Informed consent	x	x	x	x						
Allocation	x	x	x	x						
**Interventions:**										
A: Propolis (EPP-AF) + standard care										
B: Placebo + standard care										
**Assessment:**										
Baseline information	x	x	x	x						
Outcomes variables										x
Adverse events monitoring					x		x	x	x	

### Trial participants

Hospitalized patients over 18 and under 80 years of age diagnosed with SARS-CoV-2 infection, confirmed by reverse transcriptase-polymerase chain reaction, are considered eligible if symptoms started within 14 days of the randomization date. This search was actively carried out. Exclusion criteria include patients undergoing invasive oxygen therapy at the time of randomization, pregnancy or lactation, known hypersensitivity to propolis or recent use within the prior 30 days, active cancer, human immunodeficiency virus positivity, solid organ or bone marrow transplant, use of immunosuppressive drugs, bacterial or fungal infection at randomization, sepsis or septic shock, inability to administer medication orally or via a nasoenteral tube, known liver failure, advanced heart failure (New York Heart Association [NYHA] class III or IV), or end-stage kidney disease.

### Treatment groups and justification of dose used

Eligible patients are randomly assigned at a 1:1 ratio to receive Propomax® capsules produced with dehydrated standardized Brazilian green propolis extract, EPP-AF®, for 10 days at 900 mg/day (three 100-mg capsules, three times per day) or placebo (three capsules, three times per day). Hard gelatin capsules with magnesium stearate (1%), silicon dioxide (0.1%), and microcrystalline cellulose qsp (320 mg) were prepared as placebo formulations.

Both groups receive the standard treatment. The standard treatment includes supplemental oxygen, noninvasive or invasive ventilation, corticosteroids, antibiotics and/or antivirals, vasopressor support, renal replacement therapy, IABP, and ECMO, as needed.

The dose of standardized Brazilian green propolis extract was chosen based on studies that used similar doses without observing adverse effects [9–11]. The propolis extract being used in this study was prepared from a single batch to ensure for uniformity. High-performance liquid chromatography (HPLC) was performed for the batch used prior to making the capsules.

The standardized Brazilian green propolis extract, which is composed mainly of green propolis produced in southeastern Brazil and processed with a specific extraction and drying process, was selected for use in this study due to its batch-to-batch reproducibility [12, 13]. The dosage of 900 mg/day proportionally offers 47.7 mg of total flavonoids, such as quercetin and 121.5 mg of total phenolics, such as gallic acid [14, 15].

### Blinding (masking)

The study is double-blinded, so neither the patients, the health team involved in the patients’ care, nor the principal investigator know to which group the patients are allocated.

The packaging housing the capsules contains the same labeling, is opaque and has a security system to prevent improper opening. All capsules have the same organoleptic characteristics, so there is no distinction between propolis and placebo.

Decisions about standard supportive care are made by attending physicians who are not involved in the study design or randomization process.

### Randomization

To balance the distribution of oxygen support between the two groups as an indicator of severity of respiratory failure, randomization was stratified based on the following clinical parameters related to the need for supplemental oxygen: no use of oxygen, nasal catheter up to 5 L/min, nasal catheter > 5 L/min or nonrebreather mask, CPAP, or high-flow nasal cannula.

Randomization is carried out from swapped blocks listed on the REDCap platform. The permuted block (four patients per block) randomization sequence, including stratification, was prepared by a statistician not involved in the trial.

To minimize bias, concealment of allocation and drawing is performed by a trained professional who is not connected to the study. Data analysis and statistical planning are performed by an external and impartial statistician with no patient involvement. To date, there has been no interim analysis.

### Outcomes

#### Primary and secondary outcomes

The primary end point is the time to clinical improvement, defined as the difference in length of hospital stay in days between groups.

The secondary outcomes include the percentage of participants who require mechanical ventilation, secondary infection (defined when cultures of blood, urine or tracheal aspirate are positive for fungi or bacteria), acute kidney injury rate, need for renal replacement therapy, requirement for vasoactive drugs, need for IABP, or requirement for ECMO. There is a program used to analyze several interleukins before and after the intervention in some of the participants. We also assess the death rate.

Acute kidney injury is defined according to the Kidney Disease: Improving Global Outcomes (KDIGO) as stage 1 (increase in serum creatinine of 0.3 mg/dl in 48 h or increase in baseline serum creatinine by 1.5 to 1.9 times in 7 days), stage 2 (increase in serum creatinine by 2.9 times in 7 days), or stage 3 (3-fold or more increase in serum creatinine in 7 days or initiation of renal replacement therapy).

### Safety data

Safety results include adverse events that occur during treatment, serious adverse events, and premature or temporary discontinuation of treatment. Severe adverse events (pregnancy, life-threatening illness, new hospitalization, or death) are reported to the local ethics committee and the national research ethics committee.

Adverse events are classified according to the National Cancer Institute Common Terminology Criteria for Adverse Events, version 4.03.

### Data collection

Data collection is carried out through the REDCap program. The program allows for a secure and self-sufficient database and enables exportation of the data to statistical programs. The program is secure and designed for data capture with intuitive interfaces.

The data are fed and reviewed in a blinded and remote manner, ensuring accuracy and integrity according to the study’s monitoring protocol and plan.

### Participant monitoring

Patients are be monitored to ensure safety, which is the major premise of the entire study. Adverse events that may compromise patient safety or are considered serious or severe are reported to the local research ethics committee (within 24 h), an independent organization, and such events could result in interruption of the study, regardless of the study results or stage.

Patients are evaluated daily during hospitalization, from days 1 to 28. Patients who are discharged before 10 days complete treatment at home and are followed up by telephone.

The data monitoring committee (DMC), independent from the sponsor and competing interests, is responsible by the data monitoring procedure to ensure the accuracy and completeness of data reported by researchers, including information that is reported to the local ethics committee about any adverse event.

Protocol amendments require authorization from the local ethics committee; therefore, they are previously requested and independently analyzed.

### Statistical analysis

In this section, we describe the main statistical resources for this clinical trial.

#### Analysis of the primary outcome

The main analysis of the study is being conducted under the intention to treat precept.

The primary outcome of the study is defined as the length of hospital stay from truncated randomization to 28 days if the patient remains hospitalized after that period. Additionally, if the patient does not survive hospitalization, we consider a length of hospital stay equal to 28 days as the primary endpoint, even if death occurs within an interval of less than 28 days after randomization. The data for patients who cannot be reached for the 28-day follow-up are censored at hospital discharge. The between-group comparison is evaluated by generalized additive models assuming beta-binomial distribution, with adjustment by the stratification variable (type of oxygenation at baseline) and presented with 95% confidence intervals.

#### Analysis of secondary and safety outcomes

Secondary binary outcomes, such as the need for dialysis and mortality, are evaluated by logistic regression models and presented relative to chance with respective 95% confidence intervals. Continuous outcomes are described by means and standard deviations and compared by generalized regression models with distributions that best fit the data. More details are provided in the statistical analysis plan.

All secondary outcomes are adjusted by the stratification variable. Unadjusted models are subjected to sensitivity analyses.

Safety outcomes include adverse events that occur during treatment, serious adverse events, and premature or temporary discontinuation of treatment. Adverse events are classified according to the National Cancer Institute Common Terminology Criteria for Adverse Events, version 4.03.

Statistical analyses are performed using R software (R Core Team, Vienna, Austria).

### Sample size and power

The average length of hospital stay in the control group in the BeeCovid study was 12.6 days with a standard deviation of 6.5 days [2]. Based on this premise, a study with 200 individuals allocated 1:1 has a power of at least 90% to identify an average effect for 3 days of hospital stay between the propolis and placebo groups.

## Discussion

BeeCovid2 is a randomized, double-blinded, placebo-controlled study that assesses the effectiveness of standardized green propolis extract as an adjunct treatment in adults hospitalized with moderate to severe acute COVID-19. The first patient was enrolled on April 14, 2021, and the trial is scheduled for completion in July 2021.

The standardized extract of Brazilian green propolis has already undergone clinical trials evaluating its safety, drug interaction, and effectiveness in chronic and acute diseases [9–11, 16]. The standardization of the product used in this study, as well as its characterization by high-performance liquid chromatography (HPLC), guarantee uniformity and enable its use in clinical trials.

The pilot open randomized clinical trial (BeeCovid) demonstrated the safety of the two doses tested (400 mg/day and 800 mg/day), as well as the benefit in anticipating clinical recovery inferred by the reduction in hospital stay [2]. The higher dose studied was also associated with a lower rate of acute kidney injury. It is known that COVID-19-associated acute kidney injury is multifactorial and is correlated with worse outcomes. Among the mechanisms are dysregulation of the immune response, cytokine storm/inflammation, endothelial lesions (formation of microthrombi) and renal tubular lesions [1, 4]. The standardized extract of green propolis EPP-AF has already demonstrated protective efficacy in an experimental model of sepsis, with evident contributions to the protection of glomerular filtration and tubular function, reduction of cytokines and macrophage infiltration in renal tissue, immunoregulation of TLR4 and NF-κB, and reduction of lung inflammation [17].

The increase in the number of cases, as well as the greater potential for infectivity and inflammation of the new variants, has prompted the search for tools to fight a disease without a specific treatment, and that causes immunological and inflammatory challenges [1, 18]. Immunomodulation and the concomitant reduction in inflammation acting on viral targets may be a good strategy to study.

Several experimental studies have already demonstrated the effects of substances present in green propolis against pathways used by the coronavirus to enter cells, trigger exaggerated inflammatory mechanisms, and promote immune responses with disordered orchestration [4–8, 19, 20].

Although the existing evidence and initial clinical data support the safety of standardized green propolis extract, new evidence for efficacy is needed. BeeCovid2 has been designed to provide accurate efficacy data. The presentation and design of the study protocol, with the methodological and statistical details, are intended to enable the study to be conducted under guidelines with respect to research ethics and according to the recommendation for Clinical Interventional Trials (SPIRIT) guideline.

## Trial status

The study is currently underway, with respect to the entire methodological design. The BeeCovid2 clinical trial was launched in March 2021 and began on April 14, 2021. The study started at a single center at Hospital São Rafael, Salvador, Bahia, Brazil.

The study is funded by the D’Or Institute for Education and Research. Maria Emília Pedreira Freire de Carvalho Foundation (FME), a nonprofit institution, collaborated with the study. The donation of standardized extract of Brazilian green propolis (EPP-AF) and placebo was made by Apis Flora Indl. Coml. Ltda. The completion of this clinical study is scheduled for July 30, 2021.

## Data Availability

Not applicable.
